# Effect of Cold Rolling Process on Microstructure, Texture and Properties of Strip Cast Fe-2.6%Si Steel

**DOI:** 10.3390/ma11071161

**Published:** 2018-07-08

**Authors:** Yunbo Xu, Haitao Jiao, Wenzheng Qiu, Raja Devesh Kumar Misra, Jianping Li

**Affiliations:** 1 State Key Laboratory of Rolling and Automation, Northeastern University, Shenyang 110819, China; yunbo_xu@126.com (Y.X.); xqjxja@163.com (W.Q.); ljp@mail.neu.edu.cn (J.L.); 2 Laboratory for Excellence in Advanced steel Research, Department of Metallurgical, Materials, and Biomedical Engineering, University of Texas at El Paso, El Paso, TX 79968, USA; dmisra2@utep.edu

**Keywords:** cold rolling, strip casting, non-oriented silicon steel, texture, magnetic properties

## Abstract

The use of twin-roll strip casting for the preparation of non-oriented silicon steel has attracted widespread attention in recent years, but related reports are limited. In this study, both one- and two-stage cold rolling with three intermediate annealing temperatures were employed to produce strip cast non-oriented silicon steel. The evolution of the microstructure and texture through the processing routes and its effect on magnetic properties were studied. Compared with one-stage rolling, two-stage rolling increased the in-grain shear bands and the retention of Cube texture in the cold rolled sheets, thereby promoting the nucleation of favorable Goss and Cube grains and restraining the nucleation of harmful {111}<112> grains. With the increase in intermediate annealing temperature, the η-fiber texture in annealed sheets was gradually enhanced, and the average grain size was increased, leading to significant improvement of magnetic properties.

## 1. Introduction

Non-oriented silicon steels are commonly used soft magnetic materials in electrical machines which assist in the conversion between electrical and mechanical energy [1,2]. After experiencing the transition from hot-rolled materials to cold-rolled materials, the manufacturing process of non-oriented silicon steels is now well developed [3,4]. The magnetic properties of silicon steels are mainly related to the crystallographic texture and grain size of the final annealed sheets [5,6]. These microstructural characteristics of materials depend on the whole processing history, which involves casting, rolling, and recrystallization annealing. Extensive research on optimizing the processing parameters, such as hot rolling temperature and cold rolling direction and annealing rate, has been carried out to obtain favorable cube and Goss textures, and to improve magnetic properties [7,8,9]. In recent years, twin-roll strip casting technology is considered as a promising alternative for the fabrication of silicon steels with high magnetic induction [10,11,12]. Strip casting significantly simplifies the process by directly supplying the thin strip from molten steel, and also gives rise to different microstructures and texture evolution compared with conventional processes.

The present study of strip cast non-oriented silicon steel mainly focuses on the initial microstructure of as-cast strip and the evolution of several special orientations. Park et al. [13] and Liu et al. [14] reported that the initial microstructure and texture of as-cast Fe-Si alloy strips was sensitive to casting parameters, while the high superheat promoted the formation of columnar grains. Jiao et al. [15] found that the coarse-grained strip with strong {100} components contributed to high magnetic induction and low core loss compared to the fine-grained strip with strong {110}<001> (Goss) texture. It is known that the recrystallization texture is developed from the deformation microstructure, which is highly dependent on rolling history, as well as the initial microstructure [16]. However, there are few studies on the relationship between microstructural evolution and the magnetic properties of strip cast non-oriented silicon under different deformation processes.

In the present work, a Fe-2.6%Si as-cast strip produced by twin-roll strip casting was respectively processed by one-stage rolling and two-stage rolling methods in an attempt to optimize the texture and magnetic properties. During two-stage rolling, a moderate rolling reduction was selected for every stage to obtain shear bands microstructures that promoted the formation of favorable <001>//RD texture. In addition, different intermediate annealing temperatures were designed because the intermediate annealing temperature affected the final deformation microstructure and recrystallization texture. These characteristics had great influence on magnetic properties, e.g., large grain size decreased the hysteresis loss and strong {100} texture increased the magnetic induction [5,6]. The microstructure and texture evolution along the entire processing route was investigated in detail. The focus was on elucidating the effects of cold deformation and an intermediate annealing process on through process microstructure, texture evolution, and final magnetic properties of strip cast non-oriented silicon steel.

## 2. Materials and Methods

**Figure 1:**
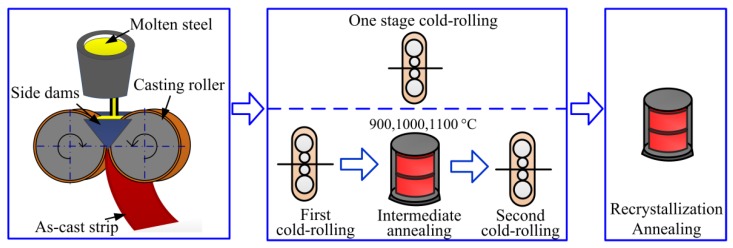
Schematic diagram of different processing routes for the as-cast Fe-Si alloy strip.

The as-cast Fe-Si alloy strip with a thickness of 2.4 mm was prepared by a laboratorial twin-roll strip caster with a melt superheat of ~50 °C. The chemical composition of the material was Fe-0.005C-2.6Si-0.2Mn-0.4Al (in wt %). The calculation by Thermo-Calc indicated that the microstructure of this material was ferrite at any temperature below liquidus, i.e., no phase transformation. Samples of size 90 mm (length) × 120 mm (width) were cut from the strip and pickled in a hydrochloric acid solution to remove the oxide scale. Subsequently, four different processes were adopted: (1) one-stage cold rolling to 0.35 mm, with final annealing at 1000 °C for 6 min, referred as OCR process; (2) two-stage cold rolling to 0.35 mm, with the first cold rolling to 0.90 mm and the intermediate annealing at 900 °C for 6 min and final annealing at 1000 °C for 6 min, referred as TCR9 process; (3) two-stage cold rolling to 0.35 mm, with the first cold rolling to 0.90 mm and the intermediate annealing at 1000 °C for 6 min and final annealing at 1000 °C for 6 min, referred as TCR10 process; (4) two-stage cold rolling to 0.35 mm, with the first cold rolling to 0.90 mm and the intermediate annealing at 1100 °C for 6 min and final annealing at 1000 °C for 6 min, referred as TCR11 process. Here, the intermediate annealing temperature of 900–1100 °C was above the recrystallization temperature of this material. The intermediate annealing schedule, including temperature and time, was designed to obtain a fully recrystallized microstructure, thereby eliminating the hereditary detrimental deformation texture of the first reduction. The annealing process was conducted in an atmosphere of pure N_2_. The schematic diagram of strip casting and different processing routes is shown in Figure 1.

The microstructure of all samples was observed using a Leica Q550IW optical microscope (OM, Leica Camera AG, Wetzlar, Germany). IPP software was used to analyze the distribution of grain size. Samples for OM observations were mechanically polished and etched with 5% nital solution. To analyze the macro-textures, the incomplete pole figures {200}, {110}, and {211} were measured on a Bruker D8 Discover X-ray diffraction (XRD, Bruker, Billerica, MA, USA) with polar angle α ranging from 0° to 75°. The ODFs (orientation distribution functions) were calculated based on three incomplete pole figures by the series expansion method (*I*_max_ = 22). Micro-textures of partially recrystallized samples were determined by a EBSD (electron backscatter diffraction) system attached to a ZEISS ULTRA 55 field emission scanning electron microscope (ZEISS, Oberkochen, Germany). Samples for XRD and EBSD analysis were electropolished beforehand with 11% perchloric acid/alcohol solution. In addition, a single sheet tester was used to measure the magnetic properties of annealed sheets, both in rolling direction and transverse direction. The magnetic induction B_50_ and core loss P_15/50_ at frequency of 50 Hz was determined at a field strength of 5000 A/m and a magnetic flux density of 1.5 T, respectively.

## 3. Results and Discussion

### 3.1. Microstructure and Texture of As-Cast Strip

**Figure 2:**
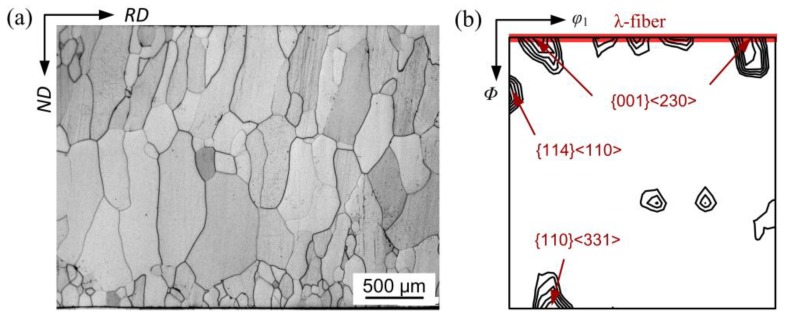
(**a**) metallographic structure of the as-cast strip and (**b**) macro-texture (φ_2_ = 45° section of ODFs) of the as-cast strip.

The microstructure and texture of the as-cast strip are given in Figure 2. The microstructure through the thickness was mainly composed of columnar grains and some equiaxed grains with grain diameters ranging from ~60 to ~770 μm; the average grain size was ~330 μm (Figure 2a). The small grains were mainly observed at the surface of strip. The texture was characterized by pronounced λ-fiber (<100>//ND) texture with strong {001}<230> orientation (Figure 2b). In addition, a few deformation components, e.g., {114}<110> and {110}<331>, were also noted. This kind of as-cast structure was similar to Liu’s observation [14], but different to the result of Park [13]. During strip casting, the heterogeneous nucleation with high nucleation rate occurred under large supercooling when molten steel met casting rollers, leading to the formation of solidified shells with fine grains at the surface of strip. Subsequently, the supercooling at the solidification front decreased, resulting in a reduction of nucleation rate. Moreover, the high superheat in this study contributed a large temperature gradient along the normal direction or heat extraction direction, which promoted the growth of columnar dendritic grains and the formation of {100} texture [15]. Before the strip left the casting rollers, the original solidification microstructure experienced slight plastic deformation, and thus, the deformation texture was developed.

### 3.2. Effect of Cold Rolling Process on Microstructure

**Figure 3:**
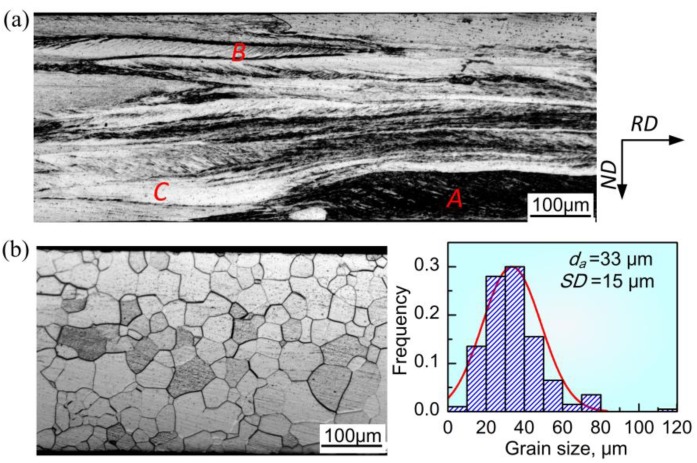
Optical microstructure of sheets processed by one-stage cold rolling. (**a**) cold-rolled sheet and (**b**) final annealed sheet.

Typical optical micrographs of cold rolled and annealed sheets processed by one stage rolling are shown in Figure 3. Three kinds of deformed microstructure corresponding to various etching degree were observed (Figure 3a): rough and darkly etched grains with high dislocation density, such as region A; moderately etched grains with in-grain shear bands, such as region B; and smooth and lightly etched grains, such as region C. As reported by Sanjari et al. [17], the smooth grains had low stored energy and were generally oriented by <100>//ND or <110>//RD textures. In contrast, the rough grains exhibited high stored energy and were usually characterized by <111>//ND texture with high Taylor factor. It can be seen that grains like those in regions A and C dominated the cold-rolled microstructure of OCR sample. Figure 3b displays the annealed microstructure; the statistical analysis of grain diameters measured from about 200 random grains using IPP. Here, *d*_a_ is average grain size, and *SD* is standard deviation to reflect the degree of grain size dispersion, i.e., uniformity. The size of grains was mainly in the range of 10–60 μm; the average value was ~33 μm with a standard deviation of ~15 μm, revealing a relatively homogeneous microstructure.

**Figure 4:**
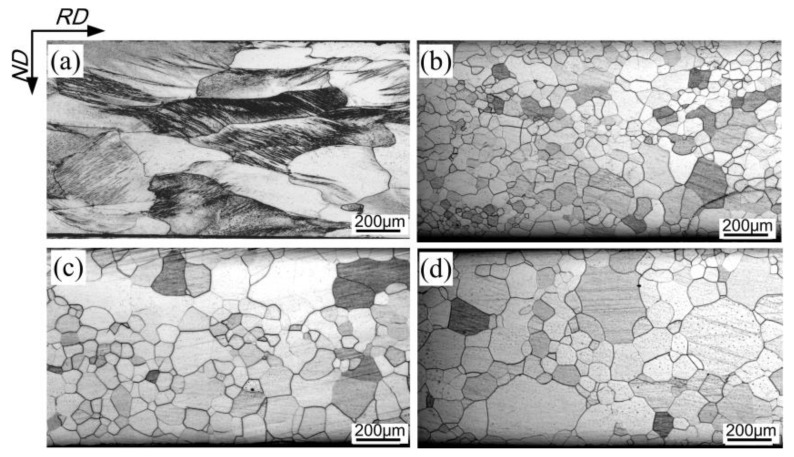
Microstructure of sheets processed by two-stage cold rolling. (**a**) First cold-rolled sheet; (**b**) 900 °C intermediate-annealed sheet; (**c**) 1000 °C intermediate-annealed sheet and (**d**) 1100 °C intermediate-annealed sheet.

**Figure 5:**
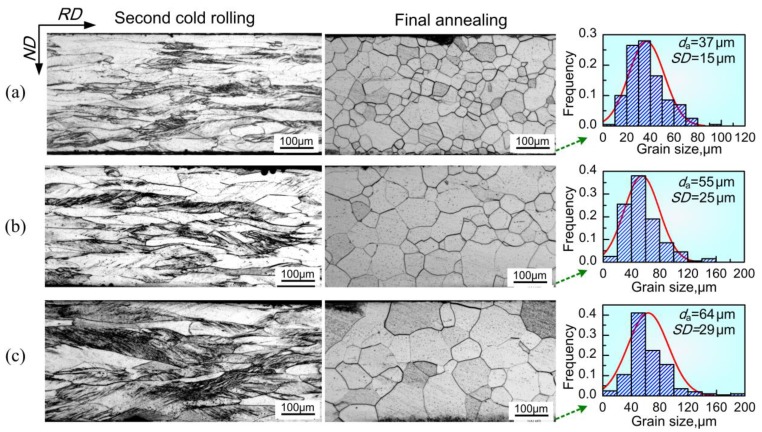
Microstructural evolution during second cold rolling and final annealing corresponding to (**a**) TCR9; (**b**) TCR10 and (**c**) TCR11 processes.

Figure 4 and Figure 5 illustrate the microstructural evolution in samples processed by two-stage cold rolling with a different intermediate annealing temperature. Compared to the deformation microstructure in the OCR process, the first cold-rolled sheet with a reduction of ~62.5% exhibited more rough grains, with a number of in-grain shear bands (Figure 4a). The microstructure was completely recrystallized after intermediate annealing. Several abnormally large grains were observed in 1000–1100 °C annealed sheets, and the average grain size increased from ~85 μm to ~120 μm with increase in annealing temperature. After second cold rolling, the majority of grains through the thickness of TCR9 sample were smooth and moderately elongated, except for the locally observed grains with high density of dislocations (Figure 5a). For TCR10 and TCR11 samples, the smooth deformation grains gradually decreased, while the fraction of shear bands gradually increased (Figure 5b,c). Furthermore, the length of shear bands was also increased. After final annealing, the average size of corresponding recrystallized grains increased from ~37 to ~64 μm, together with the deterioration of microstructural homogeneity.

It is known that dislocation slip is the main mechanism during plastic deformation, which leads to the formation of dislocation cells, dislocation walls, or microbands, depending on the imposed strain and initial orientation [18,19]. In addition, shear bands occur as a specific manifestation of local plastic instability at medium to large strains [20]. In the case of one-stage cold rolling, the shear bands that formed at moderate reduction, such as the case in Figure 4a, were destroyed during the further reduction, resulting in severely fragmented microstructure with a high density of dislocations (such as region A in Figure 3a). This kind of microstructure had high stored energy and provided a number of nucleation sites, leading to small grain size after recrystallization (Figure 3b). In the case of two-stage cold rolling, the grain size of intermediately annealed sheets and the strain during second cold rolling were the deciding factors affecting the final deformation microstructure. According to the results reported elsewhere [20,21,22], shear bands were readily developed at a reduction of 61.2% during second rolling in this study. Nevertheless, the small grains in intermediately annealed sheets of TCR9 sample restricted shear banding because of the existence of a large number of boundaries. As for TCR10 and TCR11 samples, the increased grain size in intermediate annealed sheets promoted shear localization because of weak strain coordination in grains. On the other hand, the increased grain size prior to deformation also decreased the nucleation sites, and thereby increased the size of recrystallized grains.

### 3.3. Effect of Cold Rolling Process on Texture

**Figure 6:**
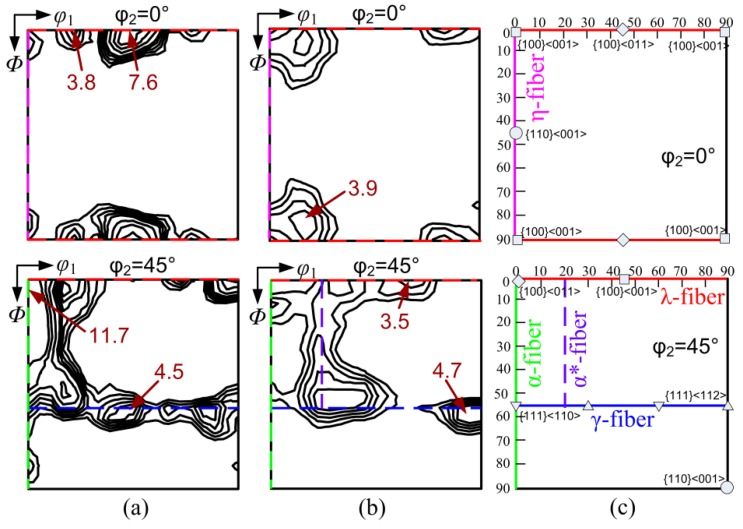
Macro-texture of OCR sample after (**a**) cold rolling and after (**b**) final annealing, and (**c**) major orientations and fibers on φ_2_ = 0° and φ_2_ = 45° sections of ODFs.

The evolution of macro-texture during different processing routes was characterized by XRD and ODF, where the orientations were expressed by Euler angles (*φ*_1_, Φ, φ_2_). In the case of OCR, the cold rolling texture was characterized by strong γ-fiber (<111>//ND) texture and α-fiber (<110>//RD) texture composed of {001}<110>–{111}<110> (Figure 6a). This belongs to the typical rolling texture in low carbon steel sheets [23]. In addition, some {001}<130> component was observed along with the <100>//ND orientation (λ-fiber) line, indicating the retention of initial {100} texture in as-cast strip. The volume fraction of {100} components was ~2.15. The maximum of *f*(g) = 11.7 was presented at (0°, 5°, 45°) close to {100}<011> orientation. After annealing, α deformation texture was rarely observed, except for a small amount of {110}<118> at (0°, 10°, 45°). The maximum orientation intensity dropped to ~4.7 at (85°, 60°, 45°). The recrystallization texture consisted of predominant {111}<112> with 5° deviation and {001}<140> components, together with weak α*-fiber ({11h}<12$\frac{1}{h}$>) texture. This kind of annealing texture in the OCR sample differed from the commonly observed in strip cast non-oriented silicon steels of 0.50 mm, which displayed strong Cube and Goss texture and weak γ-fiber texture [15,24,25]. This can be attributed to the change in recrystallization behavior due to the increase of severely fragmented grains and the decrease of shear bands in the cold rolled sheets.

**Figure 7:**
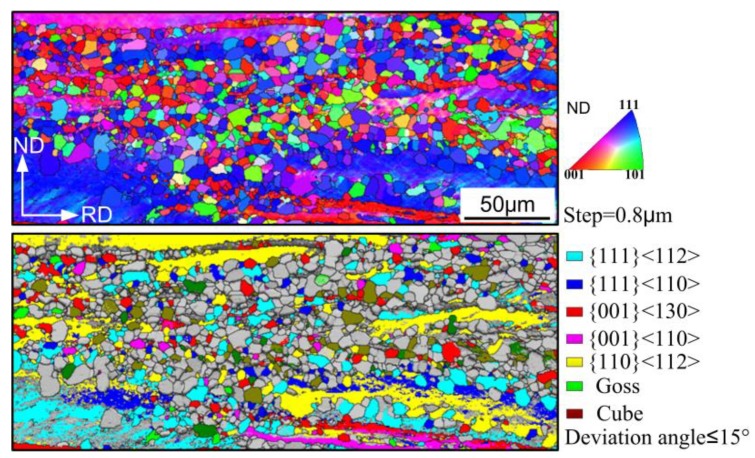
Inverse pole figure map (IPF) and relevant orientation image maps (OIM) of several main components in the partially recrystallized OCR sample.

In order to further understand the development of recrystallization texture, the micro-orientation in partially recrystallized OCR sample was measured. The corresponding IPF and OIM in Figure 7 show that a large number of grains with {111}<112> orientation preferentially nucleated in the {111}<110> and {111}<112> oriented deformation matrix, which can be explained by the oriented growth theory and oriented nucleation theory [26]. Furthermore, the recrystallized {111}<112> grains generally possessed larger size than other grains. Many {001}<140> oriented grains were also observed to be present in the microstructure. They were inherited from the characteristics of initial columnar grains with {100} texture during rolling and annealing [27]. However, the deformed {110}<112> grains were hard to recrystallize because of their lower stored energy, and were gradually consumed by new grains. It can be inferred that {111}<112> and {001}<140> grains dominated the subsequent grain growth process by virtue of nucleation and size advantage, thereby leading to the recrystallization texture pattern in OCR sample.

**Figure 8:**
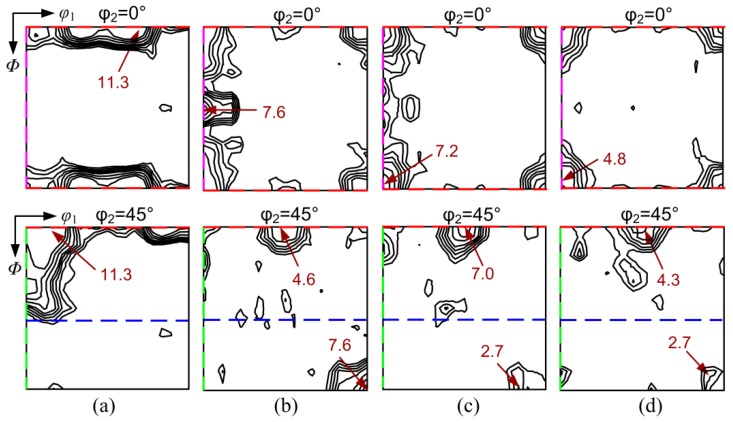
Macro-textures of (**a**) cold rolled sheet after first reduction; (**b**) 900 °C intermediately annealed sheet; (**c**) 1000 °C intermediately annealed sheet and (**d**) 1100 °C intermediately annealed sheet during two-stage rolling process.

In the case of two-stage cold rolling, the texture after first cold rolling was composed of primary α-fiber and minor λ-fiber texture (Figure 8a), where the maximum *f*(g) = 11.3 was presented at (15°, 0°, 45°) or {001}<120>. Compared with the texture of cold-rolled OCR sample (Figure 6a), relatively small deformation significantly decreased the γ-fiber texture and enhanced the retention of the initial {100} texture in first cold rolled sheets. After intermediate annealing at 900–1100 °C for 6 min, a similar texture pattern, i.e., pronounced η-fiber with peaks at Goss and Cube orientation, was developed in the three annealed samples (Figure 8b–d). However, the texture components (especially the Goss orientation) on η-fiber and the intensity were decreased with an increase in the intermediate annealing temperature.

**Figure 9:**
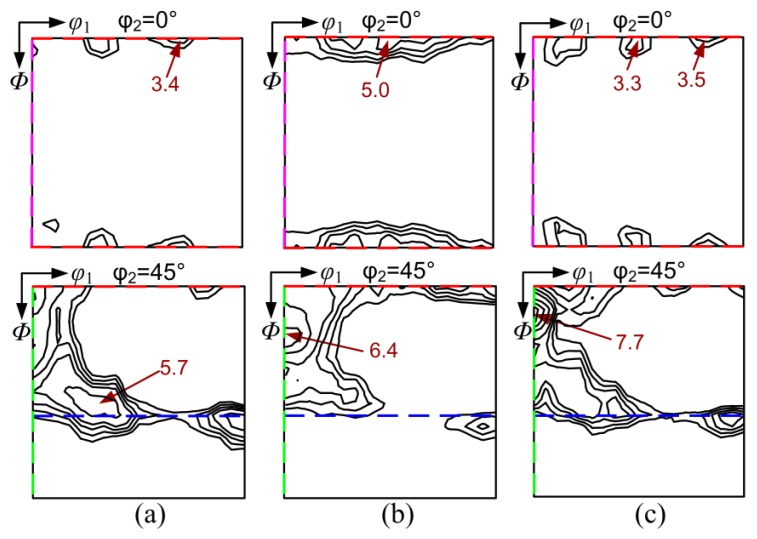
Second cold rolling textures of (**a**) TCR9 sample; (**b**) TCR10 sample and (**c**) TCR11 sample.

Figure 9 shows the texture after second rolling for the two-stage processed sample. The texture of the TCR9 sample was mainly characterized by α-fiber and γ-fiber texture with a peak at near {111}<112> orientation. In the case of TCR10 and TCR11, α-fiber was increased while γ-fiber was weakened. Meanwhile, the maximum intensity of texture was gradually increased, and the corresponding orientation shifted from {111}<112> to {114}<110>, and then to {118}<110>, which was 10° away from {100}<011>. In addition, more {100} components were observed in the latter two samples. Compared to one-stage cold rolling (Figure 6), α and γ texture in the cold rolled sheets processed by two-stage rolling was weakened, and the retention of {100} texture was enhanced because of smaller crystal rotation.

**Figure 10:**
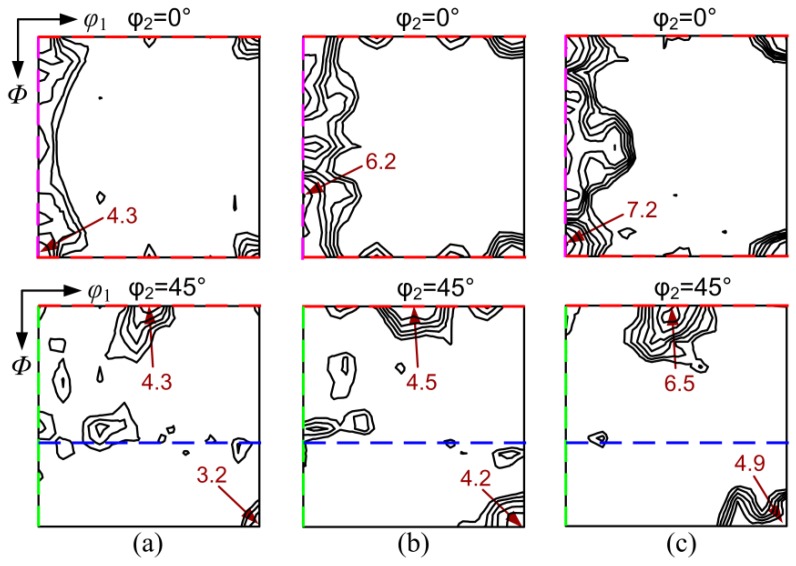
Final annealing textures of (**a**) TCR9 sample; (**b**) TCR10 sample and (**c**) TCR11 sample.

Figure 10 shows the recrystallization texture of final annealed sheets produced by two-stage rolling. All samples featured very weak γ-fiber texture and pronounced η-fiber texture with distinct Goss and Cube components, which was clearly different from the {111}<112> and {001}<140> annealing texture in the OCR sample (Figure 6). Moreover, η-fiber texture was further enhanced with increased intermediate annealing temperature. This was related to the change of deformation microstructure and texture.

**Figure 11:**
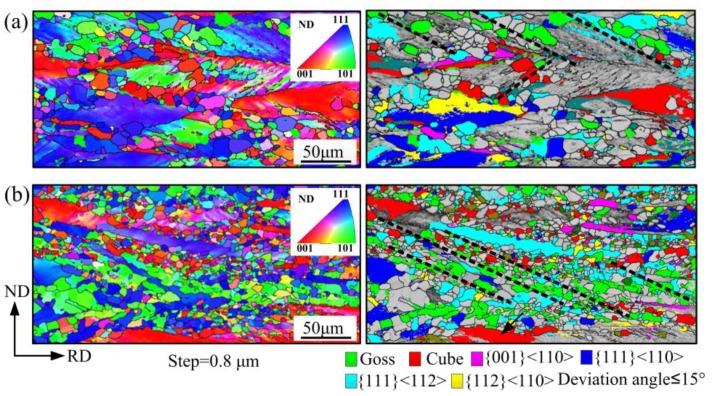
Inverse pole figure map (IPF) and relevant orientation image maps (OIM) of several main components in (**a**) partially recrystallized TCR10 sample and (**b**) partially recrystallized TCR11 sample.

The development of the recrystallization texture in two-stage rolling processed samples was related to the change of deformation microstructure and texture. Figure 11 shows the orientation image maps of partially recrystallized TCR10 and TCR11 samples. Compared with one-stage rolling, the two-stage cold rolling process significantly changed the recrystallization behavior of deformation microstructure. The {111}-oriented deformation matrix showed slow recrystallization, whereas the in-grain shear bands within these grains recrystallized rapidly. Furthermore, the grains nucleated in the {111} deformation matrix had major Goss orientation and minor Cube orientation, in accordance with other related experimental results [15,21,28]. New Goss and Cube grains gradually grew and dominated the microstructure by consuming the surrounding deformation matrix. It was noted that the nucleation density of Goss grains in the TCR11 sample was significantly higher than that of the TCR10 sample. The Goss grains were known to originate from the retention of initial Goss orientation between microbands and the newly formed Goss orientation within the shear bands in {111}<112> crystals [29,30]. Similar to Goss orientation, new Cube grains nucleated at shear bands and Cube deformation bands (the retention of Cube orientation) [31], as shown in Figure 11. The volume fraction of Goss orientation in cold-rolled TCR10 and TCR11 samples was ~1.80 and ~1.66, respectively, while that of Cube were ~3.53 and ~3.48, with small differences between the two samples. Therefore, more shear bands in the TCR11 sample were mainly responsible for the formation of more Goss and Cube nuclei. The increase in intermediate annealing temperature increased the grain size prior to final rolling, resulting in more shear bands in final cold rolled sheets. Ultimately, the η-fiber texture with sharp Goss and Cube components was gradually enhanced.

### 3.4. Magnetic Properties under Different Rolling and Annealing Processes

**Figure 12:**
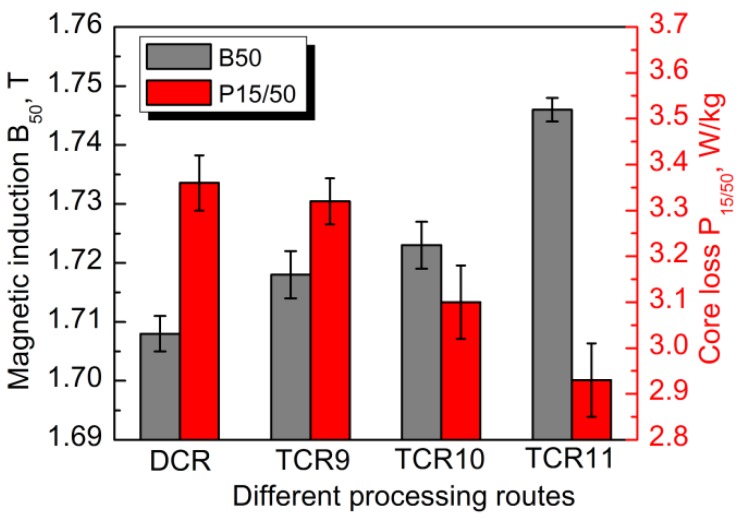
Average magnetic properties of the final anneal sheets produced by different rolling processes.

It is known that the recrystallization texture and annealing microstructure has great effect on the magnetic properties of non-oriented silicon steels. In bcc iron, <001> crystal direction is the easiest magnetization direction because of the lowest magnetocrystalline anisotropy energy, and <111> is the hardest magnetization direction [11,12]. Thus, <001>//RD (η-fiber) and <001>//ND (λ-fiber) textures are considered to be favorable for magnetic properties, while the <111>//ND (γ-fiber) texture is harmful. In addition, the total core loss of non-oriented silicon steel consists of hysteresis loss, classical eddy current loss, and anomalous loss, while hysteresis loss dominates at low frequencies [11,32]. Usually, hysteresis loss decreases with an increase in the grain size of the annealed sheets, because of a decrease in area of the domain walls. Figure 12 shows the average magnetic properties of all annealed sheets. The values of B_50_ and P_15/50_ as ~1.708 T and ~3.36 W/kg, respectively, were obtained with the DCR sample. Here, the strong {111}<112> recrystallization texture with <111> hardest magnetization direction in the DCR sample was responsible for the low magnetic induction. The high core loss was mainly related to the small grain size, even though the detrimental {111}<112> texture also increased the loss. In the case of the TCR9 sample processed by two-stage rolling, the γ-fiber texture was significantly weakened, while the λ-fiber and η-fiber textures were enhanced (Figure 10a), together with a slight increase in average grain size (Figure 5a). This suggested more <001> easy magnetization direction in rolling plane, as well as less domain walls. As a result, a slight reduction in P_15/50_, together with an increase of B_50_ by 0.01 T, was present in the TCR9 sample. When higher intermediate annealing temperatures were adopted, the magnetic properties were further improved, while the highest magnetic induction (~1.745 T) and lowest core loss (~2.92 W/kg) were observed in the TCR11 sample. This is attributed to further enhancement of λ-fiber and η-fiber textures, as well as increase in grain size. In general, two-stage rolling was an effective method to optimize the microstructure, texture, and magnetic properties of strip cast non-oriented silicon steels with thickness of 0.35 mm or less. The intermediate annealing temperature played an important role in this process. It should be noted that other processing conditions during two-stage rolling, such as the annealing atmosphere, may also affect the final texture and properties. Therefore, the best suitable set of processing conditions needs to be further studied to maximize the magnetic performance.

## 4. Conclusions

In this study, a Fe-2.6%Si-0.2%Mn-0.4%Al as-cast strip was processed by one-stage cold rolling and two-stage cold rolling, with intermediate annealing of 900–1100 °C. The evolution of microstructure and texture during different processing routes and its effect on magnetic properties were studied. The main results are listed as follows.

(1) The cold rolled sheets produced by two-stage showed significantly more in-grain shear bands compared to sheets processed by one-stage rolling. With an increase in intermediate annealing temperature, the fraction and length of shear bands in deformed microstructures, and the average grain size of final annealed sheets, was gradually increased, whereas the uniformity of the microstructure was deteriorated.
(2) Two-stage rolling weakened the γ-fiber texture and increased the retention of {100} texture in the cold rolled sheets. The annealed sheets produced by one-stage rolling exhibited a strong {111}<112> and {001}<140> texture and weak α*-fiber texture, while those produced by two-stage rolling displayed very weak γ-fiber texture and pronounced a η-fiber texture with peaks at Goss and Cube orientation, while the intensities were gradually enhanced with an increase in intermediate annealing temperature.
(3) Two-stage cold rolling was favorable to improve the magnetic properties of strip cast non-oriented silicon steel. The magnetic induction increased and the core loss decreased with the increase in intermediate annealing temperature. The best combination of B_50_ and P_15/50_ as ~1.745 T and ~2.92 W/kg was obtained when 1100 °C intermediate annealing was performed.
